# Alzheimer's disease and related dementias among transfeminine adults: A cohort study

**DOI:** 10.1002/alz.71277

**Published:** 2026-03-08

**Authors:** Ethan C. Cicero, Jace D. Flatt, Oumaima Kaabi, Vin Tangpricha, Darios Getahun, Courtney McCracken, Timothy L. Lash, Michael J. Silverberg, Suma Vupputuri, Molly Perkins, Lisa L. Barnes, Michael Goodman

**Affiliations:** ^1^ Nell Hodgson Woodruff School of Nursing Emory University Atlanta Georgia USA; ^2^ School of Public Health, Department of Social and Behavioral Health University of Nevada Las Vegas Nevada USA; ^3^ Rollins School of Public Health Emory University Atlanta Georgia USA; ^4^ Division of Endocrinology, Metabolism & Lipids, Department of Medicine Emory University Atlanta Georgia USA; ^5^ Department of Research & Evaluation Kaiser Permanente Southern California Pasadena California USA; ^6^ Department of Health Systems Science Kaiser Permanente Bernard J. Tyson School of Medicine Pasadena California USA; ^7^ Center for Research and Evaluation Kaiser Permanente Georgia, 9 Piedmont Center Atlanta Georgia USA; ^8^ Division of Research Kaiser Permanente, Northern California Pleasanton California USA; ^9^ Mid‐Atlantic Permanente Research Institute Kaiser Permanente Mid‐Atlantic States Washington District of Columbia USA; ^10^ Division of Geriatrics and Gerontology, Department of Medicine Emory University Atlanta Georgia USA; ^11^ Rush Alzheimer's Disease Center Rush University Medical Center Chicago Illinois USA

**Keywords:** Alzheimer's disease, electronic health records, gender minority, prevalence, transfeminine, transgender

## Abstract

**INTRODUCTION:**

We investigated whether Alzheimer's disease and related dementias (ADRD) are more common among transfeminine (TF) adults than among demographically similar cisgender people enrolled in the same health system.

**METHODS:**

We analyzed electronic health records of 856 TF adults aged 65+ and matched cisgender men (CM) and cisgender women (CW) and compared ADRD prevalence across groups by calculating enrollment‐adjusted odds ratios (aOR) and 95% confidence intervals (CI).

**RESULTS:**

The aOR of ADRD among TF adults were 1.39 (95% CI: 0.99–1.97) relative to CM and 1.29 (95% CI: 0.92–1.82) relative to CW referents. For TF adults with evidence of receiving gender‐affirming hormone therapy (GAHT) receipt, the associations were slightly stronger: 1.75 (1.13–2.69) and 1.70 (1.11–2.60). Results restricted to minoritized ethnoracial groups appeared smaller, but imprecise.

**DISCUSSION:**

These findings suggest that ADRD diagnosis and management may represent a priority in the healthcare of older TF people, particularly those with a history of GAHT.

## Highlights

- One of the first prevalence estimates of Alzheimer's disease and related dementias (ADRD) among transfeminine (TF) adults with a history of gender‐affirming hormone therapy receipt (GAHT).
- TF adults had a significantly higher prevalence of ADRD compared to their matched cisgender men and cisgender women referents.
- After adjusting for enrollment time, the associations were no longer significant.
- TF adults with a history of GAHT had higher ADRD odds than cisgender referents.
- Future incidence studies may help disentangle the effects of GAHT from other factors influencing ADRD risk.

## 1 BACKGROUND

Approximately 7 million adults age 65 and older are living with Alzheimer's disease and related dementias (ADRD) in the United States, and this number is expected to double by 2060.^1^, ^2^ Currently ADRD affects one in nine older adults and one out of every three die with this condition.^2^ In 2021, Alzheimer's disease, the most common cause of dementia, was the fifth leading cause of death among those age 65 and older.^3^ This descriptive epidemiology indicates considerable societal burden of ADRD in the United States, and it is likely that this burden is not distributed evenly across population subgroups. ADRD prevalence exhibits a gender gradient, with cisgender women (CW) experiencing higher rates than cisgender men (CM), potentially due to a combination of biological, social, and structural factors.^4^, ^5^ One population subgroup that has received relatively little attention in ADRD research is transgender people. Transgender people are a sizable and growing minority population that includes diverse subgroups of people whose gender identity or expression differs from the sex assigned to them at birth.^6^, ^7^, ^8^ Sex is determined and assigned at birth based primarily on the appearance of external genitalia, whereas gender identity refers to an individual's sense of femininity, masculinity, neither, both, or something else.^6^ There is considerable variation in terminology used to characterize and self‐describe transgender people.^9^ For example, transgender persons may not use the term transgender to describe their gender identity or may reject dichotomous gender categories and self‐identify as nonbinary or Two‐Spirit.

Previous research has shown a higher prevalence of ADRD among transgender adults compared with cisgender adults;^10^, ^11^, ^12^ however, findings often have not been disaggregated by transgender subgroups, leaving gaps in knowledge about subgroup‐specific burden of this condition. For example, we have a limited understanding of ADRD among transfeminine (TF) adults (individuals who identify as women or within the feminine gender spectrum and were assigned male at birth). Beyond two studies—one reporting increased odds of ADRD among TF adults compared to CM,^13^ and another showing a higher predicted probability of ADRD among TF Medicare beneficiaries compared to both CM and CW beneficiaries^14^—little is known about ADRD among TF adults using gender‐affirming hormone therapy (GAHT) or TF adults from minoritized ethnoracial groups.

To address this knowledge gap, the present study used Kaiser Permanente (KP) electronic health record (EHR) data from the Study of Transition, Outcomes, and Gender (STRONG),^15^ to compare ADRD prevalence among TF adults and demographically similar CM and CW referents. We examined ADRD prevalence among the full TF STRONG cohort (defined as members aged 65 or older at end of follow‐up or enrollment) and in two subsets of the TF cohort: (1) TF cohort members with evidence of GAHT receipt and (2) TF adults from minoritized ethnoracial groups. Each TF subset was compared to their respective matched cisgender reference groups. We hypothesized that TF cohort members overall, and particularly those from minoritized ethnoracial groups, will have a higher ADRD prevalence compared to their cisgender referents. Additionally, because exogenous estrogen therapy has shown neuroprotective effects in postmenopausal CW,^16^ explored the relationship of GAHT use with ADRD prevalence among TF cohort members.

## 2 METHODS

### 2.1 Study design

Using a subset of data from STRONG,^15^ an ongoing longitudinal EHR‐based cohort of transgender people and their age‐, race‐, and region‐matched cisgender referents enrolled in KP integrated healthcare plans, this study compared ADRD prevalence among TF adults and their CM and CW referents. As previously described,^15^ transgender status of STRONG participants was confirmed by an iterative process that relied on diagnostic codes and review of free‐text clinical notes. Cohort members were further classified as TF based on relevant specific keywords in free‐text clinical notes. The cisgender cohorts were created by matching up to 10 CM and 10 CW enrollees with each TF person, without replacement, on year of birth (within a 5‐year interval), race/ethnicity, KP site, and KP membership at index date. Index date represents the first evidence of transgender status documented in the EHR for the TF cohort member and the same date was assigned to their matched CM and CW cohort members. The algorithms used to ascertain the STRONG cohort and categorize transgender members as TF were found to be accurate.^17^ A cluster ID for each matched group (one TF member and up to 10 CM and 10 CW) was assigned to allow for stratified analyses, for example, by GAHT use, or age at index date.

**RESEARCH IN CONTEXT**

1. **Systematic review**: A systematic review was conducted using PubMed to identify recent studies examining Alzheimer's disease and related dementias (ADRD) among transfeminine adults. Only two prior studies—using Medicare data and data from the *All of Us Research* Program—have examined ADRD among transfeminine adults, with mixed findings and limited subgroup analyses.
2. **Interpretation**: Our study is one of the first to use longitudinal electronic health record data from a large integrated health system to estimate ADRD prevalence among transfeminine adults. We observed higher odds of ADRD compared to cisgender referents, particularly among those with a history of gender‐affirming hormone therapy, providing new insights into subgroup‐specific risks.
3. **Future directions**: Future research should examine incidence, timing, and mechanisms of ADRD in transgender populations, with attention to GAHT dosage, duration, and formulation, as well as intersectional factors such as race, socioeconomic status, and structural discrimination.

The original STRONG cohort, initiated in 2013, included members enrolled between January 1, 2006, and December 31, 2014, in KP health systems in Georgia (KPGA), Northern California (KPNC), and Southern California (KPSC). Since its inception, the cohort has had several updates, including the most recent update (STRONG 2.0), which was used for the current analysis. STRONG 2.0 expanded the cohort ascertainment through 2022, added an additional site at KP Mid‐Atlantic States (KPMAS), and extended follow up to June 2024. Additional cohort members were identified using (1) natural language processing‐assigned transgender status, (2) diagnostic codes or sexual orientation/gender identity data, and (3) natal sex determination. Any cohort member previously confirmed was included in STRONG 2.0.

Following cohort ascertainment, all transgender and cisgender participants were linked to disease registries, care utilization, pharmacy records, and laboratory data. All procedures for cohort ascertainment and data linkages were approved by the Institutional Review Boards at each KP site and at Emory University (coordinating center) with waivers of informed consent because all analyses used limited datasets.

### 2.2 Study population

The current study analyzed STRONG data (January 1, 2006–June 1, 2024) from TF cohort members (*n *= 856) aged 65 years or older at the study end date or end of enrollment with their matched CM (*n *= 8,560) and CW (*n *= 8,560) referents. STRONG cohort members with race or ethnicity data indicating they were Asian, Black, Native Hawaiian or Pacific Islander, American Indian or Alaska Native, multiracial, or Hispanic were classified as being from a minoritized ethnoracial group. Evidence of gender‐affirming feminizing medication use among TF cohort members was determined through EHR linkages to pharmacy records using national drug codes. Feminizing gender‐affirming medications were further classified as current treatment recommendations and historical treatment approaches (see Supplemental Table 1); TF cohort members with evidence of feminizing gender‐affirming medication receipt were classified as ever receiving GAHT. Enrollment time represents the total number of years the STRONG cohort member was enrolled in KP, which may include non‐continuous periods of coverage.

### 2.3 ADRD diagnosis

ADRD diagnoses were identified from inpatient and outpatient encounters using International Classification of Disease (ICD) codes (ICD‐9: 331.0, 290.0, 290.1, 290.2, 290.3, 290.4, 294.1, 294.2, 294.8; ICD‐10: G30, F01, F03; see Supplemental Table 2), an approach used by other researchers analyzing KP EHR data.^18^, ^19^, ^20^ Prevalent ADRD was ascertained based on any evidence of the condition, before or after the index date, and met the following criteria: (1) ADRD diagnosis was documented on two or more unique days or (2) two different ADRD diagnoses were documented on the same day.

### 2.4 Statistical analysis

All analyses were conducted using SAS, version 9.4.^21^ Baseline characteristics of TF cohort members and their matched cisgender referents were summarized as means, standard deviations (SDs), and ranges for continuous variables, and frequencies and percentages for categorical variables. Enrollment time–adjusted odds ratios (aOR) and 95% confidence intervals (CIs) were estimated using logistic regression models to compare ADRD prevalence among TF adults and their matched CM and CW referents. Since the identification of our outcome may depend on how long patients were enrolled in a KP health system, we adjusted for enrollment time to account for differing lengths of enrollment across TF cohort members and their referents. The same analyses were performed using two subsets of the TF cohort: TF adults with evidence of GAHT receipt and TF adults from minoritized ethnoracial groups. Each subset was compared to their respective cisgender reference groups.

## 3 RESULTS

Table 1 presents the demographic characteristics for 856 TF adults aged 65 years and older and their matched CM (*n *= 8,560) and CW (*n *= 8,560) referents. Most participants across all groups were enrolled in KPNC (58%) and KPGA contributed the smallest proportion of STRONG members (1.6%). At end date, the mean age of TF cohort members was 72.3 years (SD = 5.7), which was greater than their CM (66.7, SD = 7.4) and CW (67.5, SD = 7.2) referents. As expected, the racial and ethnic composition of the TF cohort was similar to that of their matched referents. The majority identified their race as White (83.4%), followed by unknown/not reported (6.4%), and Black (4.4%). Among TF adults, 9.4% identified their ethnicity as Hispanic. In total, 160 TF adults (18.7%) were classified as belonging to minoritized ethnoracial groups. Among TF cohort members, 575 (67.2%) had a documented history of GAHT. Mean enrollment time was 17.8 years (SD = 10.9) for TF adults, compared to 11.8 years (SD = 10.0) for CM and 12.2 years (SD = 10.4) for CW.

**TABLE 1:** Baseline characteristics of transfeminine cohort members aged 65 + at end date and their matched cisgender referents

Parameter	Transfeminine cohort (*n *= 856)	Cisgender men (*n *= 8,560)	Cisgender women (*n *= 8,560)
Membership site			
KPGA	14 (1.6)	140 (1.6)	140 (1.6)
KPMAS	33 (3.9)	330 (3.9)	330 (3.9)
KPNC	498 (58.2)	4,980 (58.2)	4,980 (58.2)
KPSC	311 (36.3)	3,110 (36.3)	3,110 (36.3)
Age, years at end date^1^ (mean ± SD) [range]	72.3 ± 5.7 [65–96.3]	66.7 ± 7.4 [47.2–97.7]	67.5 ± 7.2 [47.4–102.0]
< 50	0 (0)	27 (0.3)	29 (0.3)
50–60	0 (0)	1,575 (18.4)	1,305 (15.3)
61–70	411 (48.0)	4,942 (57.7)	4,967 (58.0)
71–80	386 (45.1)	1,663 (19.4)	1,888 (22.1)
> 80	59 (6.9)	353 (4.1)	371 (4.3)
Race			
Asian	37 (4.3)	378 (4.4)	369 (4.3)
Black	38 (4.4)	367 (4.3)	372 (4.4)
Multiracial^2^	12 (1.4)	97 (1.1)	102 (1.2)
Unknown/not reported	55 (6.4)	670 (7.8)	662 (7.7)
White	714 (83.4)	7,048 (82.3)	7,055 (82.4)
Ethnicity			
Hispanic	80 (9.4)	790 (9.2)	791 (9.2)
Minoritized ethnoracial group^3^	160 (18.7)	1,597 (18.7)	1,598 (18.7)
Enrollment time, years (Mean ± SD) [range]	17.8 ± 10.9 [0.1–67.7]	11.8 ± 10.0 [0–66.7]	12.2 ± 10.4 [0–65.8]

*Note*: The cisgender cohorts were created by matching up to 10 cisgender men and 10 cisgender women enrollees with each transfeminine person, without replacement, on year of birth (within a 5‐year interval), race/ethnicity, Kaiser Permanente site, and Kaiser Permanente membership at index date.

Abbreviations: KPGA, Kaiser Permanente Georgia; KPMAS, Kaiser Permanente Mid‐Atlantic States; KPNC, Kaiser Permanente Northern California; KPSC, Kaiser Permanente Southern California; SD, standard deviation.

^1^ End date = end of enrollment.

^2^ Due to cell counts fewer than 5, cohort members whose race was recorded as “American Indian/Alaskan Native” or “Native Hawaiian/Pacific Islander” were combined with the multiracial category.

^3^ Cohort members with race or ethnicity data recorded as Asian, Black, Native Hawaiian or Pacific Islander, American Indian or Alaska Native, multiracial, or Hispanic were classified as belonging to a minoritized ethnoracial group. Race and ethnicity were coded separately in the EHR. Because Hispanic ethnicity can occur within any racial group, counts for racial categories and the Hispanic category do not sum to the ‘Minoritized ethnoracial group’ total.

Table 2 presents the prevalence estimates and odds ratios of ADRD diagnosis among TF adults compared to their matched CM and CW referents. In the full cohort, ADRD prevalence was 4.8% among TF adults, compared with 2.7% in CM and 3.0% in CW referents. The unadjusted odds of ADRD were significantly higher for TF adults relative to both CM (OR = 1.81; 95% CI: 1.29–2.54) and CW referents (OR = 1.65; 95% CI: 1.17–2.31). After adjusting for enrollment time, the associations were no longer statistically significant.

**TABLE 2:** Prevalence of ADRD diagnosis in transfeminine adults relative to cisgender referent groups

ADRD prevalence comparison	Prevalence *N* (%)			Crude OR (95% CI)		Adjusted OR^a^ (95% CI)	
	TF cohort (*n *= 856)	Matched CM (*n *= 8,560)	Matched CW (*n *= 8,560)	TF versus reference CM	TF versus reference CW	TF versus reference CM	TF versus reference CW
Full cohort	41 (4.8)	232 (2.7)	254 (3.0)	1.81 (1.29–2.54)	1.65 (1.17–2.31)	1.39 (0.99–1.97)	1.29 (0.92–1.82)
Evidence of GAHT receipt^b^	TF with history of GAHT (*n *= 575)	Matched CM (*n *= 5,750)	Matched CW (*n *= 5,750)	TF versus reference CM	TF versus reference CW	TF versus reference CM	TF versus reference CW
	27 (4.7)	127 (2.2)	133 (2.3)	2.18 (1.43–3.34)	2.08 (1.36–3.18)	1.75 (1.13–2.69)	1.70 (1.11–2.60)
Cohort members from minoritized ethnoracial groups^c^	TF from minoritized ethnoracial groups (*n *= 160)	Matched CM (*n *= 1,600)	Matched CW (*n *= 1,600)	TF versus reference CM	TF versus reference CW	TF versus reference CM	TF versus reference CW
	5 (3.1)	36 (2.3)	49 (1.5)	1.40 (0.54–3.62)	1.02 (0.40–2.60)	1.23 (0.47–3.21)	0.89 (0.35–2.27)

*Note*: Each transfeminine adult was matched to 10 cisgender men and 10 women on birth year, race/ethnicity, study site, and index date (first evidence of transgender status). International Classification of Disease (ICD) codes examined for ADRD, ICD‐9: 331.0, 290.0, 290.1, 290.2, 290.3, 294.1, 294.2, 294.8, 290.4; ICD‐10: G30, F01, F03. ADRD diagnosis inclusion criteria: (1) ADRD diagnosis on two or more unique days (can be the same diagnosis on multiple days), (2) two different ADRD diagnoses on same day.

Abbreviations: ADRD, Alzheimer's disease and related dementias; CI, confidence interval; CM, cisgender men; CW, cisgender women; GAHT, gender‐affirming hormone therapy; OR, odds ratio; TF, transfeminine.

^a^ Adjusted for enrollment time.

^b^ Analyses restricted to those transfeminine cohort members with a history of GAHT and their matched cisgender referents.

^c^ Analyses restricted to transfeminine cohort members from minoritized ethnoracial groups and their matched cisgender referents.

The prevalence of ADRD among TF individuals with a history of GAHT was 4.7%, compared to 2.2% in CM and 2.3% in CW referents. In analyses restricted to TF adults with evidence of GAHT and their cisgender referents, the odds of ADRD remained significantly elevated. The strength of these associations was slightly greater than those observed in the crude models of the overall cohort (versus CM: OR = 2.18; 95% CI: 1.43–3.34 and versus CW: OR = 2.08; 95% CI: 1.36–3.18). After adjusting for enrollment time, the associations remained significant, though the magnitude was attenuated relative to both CM (aOR = 1.75; 95% CI: 1.13–2.69) and CW referents (aOR = 1.70; 95% CI: 1.11–2.60).

Among TF cohort members from minoritized ethnoracial groups, ADRD prevalence was 3.1%, compared to 2.3% in CM and 1.5% in CW referents. The odds of ADRD among TF adults was not appreciably different from either CM or CW referents in unadjusted or adjusted analyses. However, these results were imprecise due to the small numbers of ADRD cases in this group.

## 4 DISCUSSION

This study builds on the limited but growing body of research examining ADRD in transgender populations by providing one of the first prevalence estimates of ADRD among TF adults with a history of GAHT and those from minoritized ethnoracial groups, using EHR data from a large, multi‐site integrated health system. Consistent with our hypothesis, TF adults had a significantly higher prevalence of ADRD compared to their matched CM and CW referents, but after adjusting for enrollment time, the associations were no longer significant, though the 95% CI ranged from 0.99 to 1.97 for the TF versus CM comparison and 0.92 to 1.82 for the TF versus CW comparison. Restricting the analysis to TF adults with a documented history of GAHT revealed a sustained and statistically significant association with ADRD, with effect sizes modestly exceeding those observed in the overall cohort. While adjusting for enrollment time reduced the strength of these associations, they remained significant relative to both CM and CW referent groups. Among TF adults from minoritized ethnoracial groups, ADRD prevalence was low and not appreciably different from that of cisgender referent groups, although these results were imprecise due to small case counts. Of note, the age distribution of the STRONG 2.0 cohort is younger than traditional aging cohorts, with most participants in their late sixties to early seventies at the end of follow‐up. Because ADRD prevalence rises sharply in adults over age 75, this younger age profile likely contributed to the low number of ADRD cases observed in both TF and cisgender groups. As a result, prevalence estimates should be interpreted cautiously, although the matched design ensures that our relative comparisons are not biased by these age differences.

Our findings are consistent with prior research. One study using 2009–2017 Medicare fee‐for‐service data examined nearly 10,000 age‐entitled transgender beneficiaries (ages 65 years and older) and a 5% random sample of cisgender beneficiaries. After adjusting for age, race/ethnicity, census region, and months of enrollment, the predicted probability of ADRD among TF Medicare beneficiaries was about 20%, higher than the corresponding probabilities of 12% among CM and 14% among CW.^14^ Similar to the STRONG cohort, transgender status in this study was identified using a combination of transgender‐related diagnosis and procedure codes, including endocrine not otherwise specified (NOS) diagnoses in conjunction with gender‐affirming procedures or hormone prescriptions. A second study,^13^ using EHR and survey data from the *All of Us Research Program*, offers partial comparability. In that study, TF individuals were divided into two groups: those who self‐identified as transgender and those who could be characterized as gender diverse (e.g., genderqueer, genderfluid, gender variant, two‐spirit, questioning, or unsure). The gender diverse group in that study had a 2.5‐fold higher odds of ADRD relative to cisgender individuals. In contrast, the corresponding odds of ADRD were 20% lower in TF persons who identified as transgender relative to cisgender individuals. The results from the *All of Us Research Program* may not be directly comparable to our findings because our study employed different operational definitions of transgender status and used separate CM and CW reference groups.

Importantly, both previous studies applied a broader set of ICD codes to identify ADRD, including codes not typically used in ADRD research.^22^, ^23^, ^24^ While this approach may enhance diagnostic sensitivity, it complicates comparisons across studies. The present study used a more specific and commonly accepted set of codes aligned with prior epidemiologic research.^20^, ^25^, ^26^ As such, the inclusion of less commonly used codes in the other studies may partially account for their higher observed prevalence and limits the direct comparability of findings.

The observation that the odds of ADRD among TF adults with a history of GAHT may be somewhat higher than in the overall cohort is noteworthy. This finding challenges assumptions drawn from studies among CW, which suggests that exogenous estrogen therapy may offer neuroprotective benefits.^16^, ^27^ However, evidence in this area remains mixed^27^, ^28^, ^29^ and largely unexamined among TF individuals. In the present study, the aOR for the GAHT subgroup were consistently higher than those for the overall cohort and became moderately stronger in the subset of GAHT users. Given the cross‐sectional nature of this study, we cannot determine whether the higher prevalence of ADRD among TF individuals is attributable to GAHT use. Results from a related study that examined cognitive functioning among older TF individuals receiving long‐term GAHT found no significant association between GAHT duration and cognitive functioning.^30^ However, in the current study, GAHT use was defined broadly as lifetime exposure to feminizing medications. More nuanced investigations into age at initiation, dosage, route, duration, formulation of GAHT, and hormone levels are needed to better understand the relationship between GAHT and cognitive outcomes. Future incidence studies may help disentangle the effects of GAHT from other factors influencing ADRD risk.

The lack of significant findings among TF cohort members from minoritized ethnoracial groups should be interpreted with caution. The observed low ADRD prevalence in this group likely reflects limitations in case identification due to small sample sizes rather than a true absence of disparities. ADRD is well‐established as a health disparity for some racial and ethnically minoritized groups,^31^, ^32^, ^33^ and transgender adults have higher rates of ADRD rick factors such as depression, social isolation, adverse childhood experiences, and subjective cognitive decline compared to cisgender adults.^10^, ^34^, ^35^, ^36^, ^37^, ^38^, ^39^, ^40^ Future research with larger samples is needed to meaningfully examine how the intersection of race, gender identity, systemic marginalization, and transgender‐related psychosocial stressors such as anti‐transgender discrimination and unaffirming responses from family members, friends, and co‐workers may contribute to ADRD.

### 4.1 Limitations

Several limitations should be considered when interpreting these findings. This cross‐sectional analysis of prevalent ADRD does not allow for causal inferences or assessment of incidence or age at onset, and future longitudinal studies are needed to better understand the temporal relationship between gender identity, GAHT, and incident ADRD. Additionally, ADRD diagnoses were identified using diagnostic codes from EHR data, which may result in underascertainment or misclassification of cases. Nonetheless, given the higher observed prevalence of ADRD among TF individuals than matched cisgender cohorts, this differential underascertainment does not explain the observed results. This limitation may contribute to underreporting of ADRD among TF individuals, who have historically faced, and continue to experience, barriers to diagnosis and care.

Although this study leveraged a large and diverse sample from multiple KP sites, the generalizability of findings may be limited to insured individuals receiving care in integrated health systems. Additionally, because population‐level data on TF adults do not exist, we were unable to determine whether the TF sample identified at each KP site is representative of the broader state or regional TF population. Similarly, due to the relatively small number of ADRD cases among TF adults from minoritized ethnoracial groups, results for this subgroup were imprecise.

A limitation of STRONG data is the vastly different demographic and enrollment characteristics of persons with and without evidence of GAHT receipt. In addition, while the pharmacy data offer high level of confidence of GAHT use, those without filled prescriptions may still be receiving GAHT; this may be especially true for older cohort members who initiated GAHT or disenrolled from the participating plans 10 or more years ago. For these reasons, we tested the hypothesis about the potential association between history of GAHT receipt and ADRD prevalence indirectly (i.e., by comparing TF cohort members with and without evidence of GAHT use to their respective matched reference groups) and by qualitatively evaluating the difference between the two OR and aOR estimates. A direct examination of this association would require a longitudinal analysis of ADRD incidence, rather than prevalence, following GAHT initiation. Even more informative would be an assessment of the association between sex hormone levels and ADRD incidence. Unfortunately, these approaches are not feasible at this time because the number of newly diagnosed ADRD cases in the STRONG cohort is very small.

Another important limitation is that GAHT use was defined broadly as lifetime exposure to feminizing medications. Data on the specific age at initiation, dosage, route, duration, formulation, and serum hormone levels were not examined, limiting the ability to evaluate more nuanced associations between GAHT and ADRD risk. Lastly, a key limitation of this study is its exclusive focus on TF adults. Although examining ADRD prevalence among transmasculine adults and comparing them to cisgender referent groups represents an important area for future research, the number of ADRD cases among transmasculine cohort members in the STRONG dataset was insufficient to support robust statistical analyses

## 5 CONCLUSION

This study contributes evidence to the limited research on ADRD among transgender populations, demonstrating that TF adults with a documented history of GAHT have significantly higher odds of ADRD compared to demographically similar CM and CW. Future research should explore the nuanced use of GAHT and serum hormone levels in cognitive aging outcomes. To better understand and address ADRD risk and disparities, longitudinal studies are needed to investigate modifiable risk factors for ADRD that may be unique to or more prevalent among transgender populations. Identifying such factors is critical for informing clinical strategies aimed at preventing or delaying ADRD onset. As the transgender population continues to age, ensuring that ADRD research, prevention, and care efforts are inclusive of transgender populations is essential to advancing health equity across the lifespan.

## CONFLICT OF INTEREST STATEMENT

In addition to the funding sources acknowledged, the authors report no other financial or non‐financial conflicts of interest. Author disclosures are available in the Supporting Information.

## CONSENT STATEMENT

This study involved a secondary analysis of de‐identified EHR. As no direct contact with human subjects occurred and no identifiable information was used, informed consent was not required.

## Supporting information



Supporting Information

Supporting Information

Supporting Information
