# Supplementary material for: Alzheimer's disease and related dementias among transfeminine adults: A cohort study
Source: Alzheimers Dement. 2026 Mar 8;22(3):e71277. doi: 10.1002/alz.71277 (PMC12967476; doi:10.1002/alz.71277)
Supplement: Supplementary file 1 — Supporting Information [file ALZ-22-e71277-s003.docx]

| **Supplement Table 1. Feminizing gender-affirming medications for transfeminine individuals** | |
| --- | --- |
| **Current treatment recommendations*** | **Historical treatment approaches** |
| drospirenone and ethinyl estradiol, esterified estrogens, estradiol, estradiol and lidocaine in petrolatum, estradiol levonorgestrel, estradiol norethindrone, estradiol norethindrone acetate, estradiol valerate, estrogens, ethinyl estradiol, | conjugated estrogens, conjugated estrogens and bazedoxifene, conjugated estrogens and medroxyprogesterone, esterified estrogens and methyltestosterone, ethynodiol diacetate and ethinyl estradiol, etonogestrel ethinyl estradiol, hydroxyprogesterone caproate, levonorgestrel and ethinyl estradiol, medroxyprogesterone, medroxyprogesterone and estradiol cypionate, methyltestosterone, norethindrone, norethindrone acetate, norethindrone acetate and ethinyl estradiol, norethindrone and ethinyl estradiol, norethindrone and mestranol, norgestimate ethinyl estradiol, progesterone, progesterone micronized |
| Notes: *World Professional Association for Transgender Health Standards of Care, Version 8. | |
