# Supplementary material for: Alzheimer's disease and related dementias among transfeminine adults: A cohort study
Source: Alzheimers Dement. 2026 Mar 8;22(3):e71277. doi: 10.1002/alz.71277 (PMC12967476; doi:10.1002/alz.71277)
Supplement: Supplementary file 2 — Supporting Information [file ALZ-22-e71277-s001.docx]

| **Supplement Table 2. ADRD diagnostic codes from inpatient and outpatient encounters** | |
| --- | --- |
| **ICD code** | **Description** |
| ICD-9 |  |
| 331.0 | Alzheimer’s disease |
| 290.0 | **Senile dementia, uncomplicated** |
| 290.1 | Presenile dementia |
| 290.2 | Senile dementia with delusional or depressive features |
| 290.3 | **Senile dementia with delirium** |
| 290.4 | Vascular dementia |
| 294.1 | **Dementia in other specified conditions classified elsewhere** |
| 294.2 | **Dementia, unspecified** |
| 294.8 | Other persistent mental disorders due to conditions classified elsewhere |
| ICD-10 |  |
| G30 | Alzheimer’s disease |
| F01 | Vascular dementia |
| F03 | **Dementia, unspecified** |
| *Notes*: ICD: International Classification of Disease | |
